# Knowledge and practice of cervical cancer screening and associated factors among reproductive age group women in districts of Gurage zone, Southern Ethiopia. A cross-sectional study

**DOI:** 10.1371/journal.pone.0238869

**Published:** 2020-09-18

**Authors:** Daniel Adane Endalew, Dureti Moti, Nuru Mohammed, Sebaba Redi, Biresaw Wassihun Alemu

**Affiliations:** 1 Department of Midwifery, College of Medicine and Health Sciences, Wolkite University, Wolkite, Ethiopia; 2 Department of Midwifery, College of Medicine and Health Sciences, Arba Minch University, Arba Minch, Ethiopia; Makerere University School of Public Health, UGANDA

## Abstract

**Background:**

Cervical cancer is a malignant tumor of the lower-most part of the uterus and major cause of morbidity and mortality among women’s in the world. Its high mortality rate in the globe can be reduced through comprehensive approaches’ that include; primary prevention, early diagnosis, effective screening, and treatment packages. This study was aimed to assess the knowledge and practice of cervical cancer screening and its associated factors among reproductive age group women in districts of Gurage zone, Southern Ethiopia, 2019.

**Methods:**

A community-based cross-sectional study design was conducted from March 1–30, 2019. A total of 268 respondents were selected using a systematic sampling technique. Data was collected using pretested, semi-structured, and interviewer-administered questionnaires. Data were entered into Epi data version 3.1software and exported to SPSS 24 for analysis. Bivariate and multivariate analyses with a 95% confidence level was done and variables (P <0.05) were deemed statistically significant.

**Result:**

A total of 260 respondents participated in the study with a response rate of 97%. About 3.8% of the respondents had experiences cervical cancer screening and 26.2% of respondents had good knowledge. Early age at first sex [AOR = 6.05 (95%CI; 1.167–31.36)], having information about cervical cancer [(AOR = 10.2 (95% CI 1.9–96.4)], and multiple sexual partners [AOR = 3.96 (95% CI; 1.48–10.58)] were factors affecting the practice of cervical cancer screening. Being uneducated [AOR = 15.5 (95%CI; 3.82–62.967)], family history of cervical cancer [AOR = 14.158 (95%CI;3.88–51.7)], having plans to screen for cervical cancer [AOR = 0.352 (95%CI;.175-.710)], menarcheal age [AOR = 2.63 (95%CI;1.28–5.37)] and age at first sex [AOR = 3.17 (95%CI;1.283–7.837)] were factors affecting knowledge of cervical screening.

**Conclusion:**

The study findings indicate that respondents’ practice and knowledge of cervical cancer is mainly affected by early age at first sex, having information about cervical cancer, multiple sexual partners, Educational status, family history of cervical cancer, having plans to screen for cervical cancer, age at first sex and age of menarche. Therefore, all concerned bodies need to focus on women in the reproductive age group to increase the level of knowledge and practice of cervical cancer screening through appropriate interventions.

## Introduction

Cervical cancer is a cancer of the lower-most part of the uterus, which can be prevented by early detection using several diagnostic methods and Human Papilloma Virus (HPV) vaccine [1]. Its high mortality rate in the globe can be reduced through comprehensive approaches’ that include primary prevention, early diagnosis, effective screening options (Pap smear, HPV-DNA testing, and visual inspection with acetic acid and iodine) and treatment packages [1, 2]. Even when an accurate and cost-effective method for screening is available, the utilization of services is not nearly optimal.

Lack of knowledge about the disease process, inadequate pap smear testing, and the client’s negative attitude towards the procedure were the major factors associated with cervical cancer screening. Besides poor knowledge, lack of awareness of available screening methods can also play a great role not to use cervical cancer screening services [3–5].

Cervical cancer is the fourth most frequent cancer which affects 6.6% of all women in the world. It is more prevalent in low-and middle-income countries which showed that approximately 90% of women’s deaths were due to cervical cancer [6]. Cervical cancer is the second most common cancer which accounts for 22% of all female cancers and 12% of all newly diagnosed cancers every year, and the leading cause of cancer death in African women [7, 8]. For the reason that low levels of awareness, poor knowledge, and clients with the inaccessibility of services there is a low level of cervical cancer screening in sub-Saharan Africa (SSA) and other developing countries [9]. In Ethiopia, cervical cancer is the most frequent cancer among women between 15 and 44 years of age and about 35% of patients who admitted was due to cervical cancer [10, 11].

According to world health organization (WHO), screening of cervical cancer should be started at the age of 30 years and beyond. Whereas, United States’ standards recommended women must have cervical cancer screening at the age of 21 years and should be screened at least every two years for low resource settings [9, 12].

Despite universal screening is important in Ethiopia, screening is mostly conducted if a woman seeks medical care for other reasons or only if the woman presents with symptoms. Even though few studies were conducted in Ethiopia, data on knowledge and practice of cervical cancer screening and its contributing factors about cervical cancer screening in the study setting is limited. Therefore, this study aimed to assess the knowledge and practice of cervical cancer screening service and associated factors among reproductive age group women in Southern Ethiopia.

## Materials and methods

### Study design and setting

A community-based cross-sectional study design was conducted from May 1–30, 2019 in districts of Gurage zone, Southern Ethiopia. This zone is located 158 kilometers far from Addis Ababa (the capital city of Ethiopia) and 337 kilometers far from Hawassa (the capital city of South Nations Nationalities and Peoples Region). It is bordered Southwest by Hadiya and Yem special woreda, on the West, North, and East by Oromia Region, and on the Southeast by Silte zone. Wolkite is the administrative center of the zone. According to the 2007 national household census, the zone has 13 districts and two administrative towns with a total population of 1,279,646 among these 657,568 were women.

### Populations

The study populations were all reproductive age group women living in selected districts of the zone during the study period.

### Sample size determination

The sample size was determined by using the single population proportion formula by considering the following assumptions; Proportion of women knowing about cervical cancer screening of 21% (P = 0.21) [13], 95% confidence interval, the margin error of 5% (d = 0.05) and 5% non-response rate. The final sample size was adjusted to 268 study participants.

### Sampling technique and procedures

From 13 districts and 2 administrative towns, 5 districts, and one administrative town with having a total of 1,340 reproductive age group women were selected randomly. To select study participants systematic random sampling technique was used. Those eligible participants who didn’t avail during the data collection period in selected Kebele were revisited three times. To select a total of 268 respondent’s proportional allocation to population size was used in each Kebele.

### Operational definitions

Study participants who scored ≥mean were considered as having good knowledge of cervical cancer screening. Whereas, study participants who scored below the mean score of cervical cancer screening service-related questions were considered as having poor knowledge. For practice questions, respondents were asked whether they had gone for any screening test at least once or had not screened in the past 3 years. Those who had gone for screening were considered to have practiced cervical cancer screening. Those who had not gone for screening were considered as not having practiced cervical cancer screening.

### Data collection tools

An English semi-structured interviewer-administered questionnaire adapted from other existing tools was contextualized and pretested for its reliability. The content validity of the questionnaire was reviewed by qualified obstetricians and public health specialists. The survey tool and interview guides were developed by reviewing different works of literature, current national and international guidelines on cervical cancer screening. The tool consisted of socio-demographic characteristics, knowledge, the practice, reproductive history, and the lifestyle and sexual behavior of respondents. All reproductive age group women who were available in the study settings were included in the study. Four certified clinical nurses and two Bachelors of Science in nursing were recruited to support data collection.

### Data quality control

To ensure quality, the questionnaire was translated into the local language by experts. Finally, before data collection, it was re-translated back to English to verify consistency. Before starting the actual data collection, one day of training was given for the data collectors and supervisors. A pre-test was conducted on 14 (5%) of the total sample size at Wolkite town 01 Kebele by data collectors and all necessary amendments were done accordingly. The reliability of the questionnaires was checked through SPSS by reliability index measurement for practice and knowledge questions (Cronbach’s alpha) which was 0.76 and 0.78 respectively. During data collection, data collectors were supervised by field supervisors with overall activities being controlled by the principal investigator. After data collection before analysis, all collected data were checked for completeness.

### Data processing and analysis

Data entry was done by using EPI Data 3.1 and exported to SPSS version 24 for analysis. Frequency, percentage, and the mean for independent and dependent variables were computed using descriptive statistical analysis. Binary logistic regression analysis was used to ascertain the association between explanatory and outcome variables. Significant (P< 0.25) variables in bivariate analysis were entered into multivariate analysis and statistical significance was set at a p-value of <0.05. The odds ratio determined with a 95% confidence level was used to assess the strength of association. Multicollinearity was checked to see the linear correlation among the independent variables by using standard error. Variables with a standard error of > 2 were dropped from the multivariate analysis. The result of the study was presented in tables, figures, and texts.

### Ethical approval and consent to participant

Ethical clearance for the proposed study was obtained from the institutional review board of Wolkite University College of Medicine and Health Sciences. Communication with the different Town and kebeles administrators was made through a formal letter obtained from Wolkite University. After the purpose and objective of the study have been explained, informed written consent was obtained from each study participant.

## Results

### Socio-demographic characteristics of respondents

From a total of 268 respondents who were invited in the study, 260 participated in interview with a response rate of 97%. The age of respondents ranges from 15–49 with a mean and SD of 33.6 ± 7.15 years respectively. More than two-thirds (81.5%) of respondents were educated; from this 42.7% were educated up to college and above level. The majority, 48.5% of respondents were housewives (Table 1).

**Table 1:** Socio-demographic characteristics of respondents among reproductive age group women in districts of Gurage zone, Southern Ethiopia, 2019.

Variables	Category	Frequency	Percentage
The age group of participants	15–24	24	9.2
	>24	236	90.8
Marital status	Single	46	17.7
	Married	194	74.6
	Divorced	16	6.2
	Widowed	4	1.5
Ethnicity	Gurage	192	73.8
	Oromo	19	7.3
	Amhara	20	7.7
	Kembata	26	10.0
	Tigrie and Wolaita	3	1.2
Religion	Orthodox Christian	62	23.9
	Protestant	51	19.6
	Muslim	46	17.7
	Catholic	101	38.8
Work/Occupation	Farmer	25	9.6
	Housewife	126	48.5
	Government Employee	68	26.1
	Merchant	35	13.5
	Daily worker and students	6	2.3
Monthly income	0–1650 Ethiopian birr	110	42.3
	1651–3145	136	52.3
	3146–5195	13	5.0
	>5195	1	0.4
Educational status	Illiterate	48	18.5
	Primary	34	13.0
	Secondary	67	25.8
	College and above	111	42.7

### Reproductive characteristics of respondents

The majority (45.8%) of respondents had a regular menstrual history. The majority, 57.3% of respondents had started menses at the age of ≤ 12years. More than two-thirds (74.8%) of respondents had started sexual intercourse at the age group of 18–22 years. Among the respondents, 11.5% had a history of abortion and 8.5% had a family history of cervical cancer (Table 2).

**Table 2:** Reproductive characteristics of respondents among reproductive age group women in districts of Gurage zone, Southern Ethiopia, 2019.

Variables	Category	Frequency	Percentage
Age of Menarche	9–12 years	149	57.3
	13-15years	111	42.7
Sex condition	Yes	246	94.6
	No	14	5.4
Age at first sex	15–17 years	35	14.2
	18–22 years	184	74.8
	23–26 years	27	11.0
History of post-coital bleeding	Yes	32	12.3
	No	228	87.7
Contraceptive history	Yes	138	53.1
	No	122	46.9
Type of family planning used	Pills	24	17.4
	Injectable	52	37.7
	Implants	41	29.7
	Iintrauterine contraceptive device	9	6.5
	Condom	12	8.7
Nature of menses	Regular	119	45.8
	Sometimes irregular	109	41.9
	Always irregular	17	6.5
	No menses	15	5.8
History of abortion	Yes	30	11.5
	No	230	88.5
Family history of cervical cancer	Yes	22	8.5
	No	238	91.5

### Knowledge of cervical cancer screening

The majority (83.8%) of respondents had heard about cervical cancer. About 76.9% of respondents didn’t know any cervical cancer symptoms. Whereas 8.8%, 5.0%, 5.0%, and 0.4% of respondents believed that having multiple sexual partners, initiation of sexual intercourse at an early age, cigarette smoking, and acquiring human papillomavirus (HPV) respectively were the major risk factors for cervical cancer. All most all (97.7%) of the respondents didn’t know any methods of cervical cancer screening. The majority (56.0%) of respondents have acquired information about cervical cancer screening from mass-medias (Table 3). The mean score and standard deviation of respondents’ knowledge about cervical cancer screening were 39.38 and ± 7.788 respectively. The result revealed that; 26.2% of respondents were knowledgeable on cervical cancer screening.

**Table 3:** Knowledge of cervical cancer screening among reproductive age group women in districts of Gurage zone, Southern Ethiopia, 2019.

Variables	Category	Frequency	Percentage
Have you heard of cervical cancer	Yes	218	83.8
	No	42	16.2
Source of information	From media	122	56.0
	Broachers and printed materials	24	11.0
	Health workers	17	7.8
	Family, friends, neighbors	55	25.2
Cervical cancer symptoms	Vaginal bleeding	30	11.5
	Vaginal foul-smelling discharge	27	10.4
	I don’t know	200	76.9
	Post-coital bleeding	3	1.2
Cervical cancer risk factors	Having multiple sexual partners	23	8.8
	Early age initiation of sexual intercourse	13	5.0
	Cigarette smoking	13	5.0
	Acquiring human papillomavirus	1	0.4
	I do not know	210	80.8
How can cervical cancer be prevented	Through minimizing multiple sexual partners	24	9.2
	Through avoiding early sexual intercourse	9	3.5
	Through abandon smoking	16	6.2
	Through vaccination of Human Papilloma Virus	1	0.4
	I do not know	210	80.8
Can cancer of the cervix be cured at the first stage	Yes	38	14.6
	No	2	0.8
	I do not know	220	84.6
How can someone with cancer of the cervix be treated	Use of Herbal remedies	22	8.5
	Surgery	12	4.6
	Use of specific drugs given by the hospital	6	2.3
	Radiotherapy	10	3.8
	I do not know	210	80.8
How expensive do you think cancer of the cervix treatment is in this country	It is free of charge	28	10.8
	It is reasonably priced	7	2.7
	Moderately expensive	17	6.5
	It is very expensive	78	30.0
	I do not know	130	50.0
Do you know that there exists cervical cancer screening	Yes	135	51.9
	No	125	48.1
How frequent is screening for cervical cancer performed	Once every year	2	0.8
	Once every three years	11	4.2
	Once every 5 years	5	1.9
	1 do not know	242	93.1
Can you mention any of the procedures used in screening for cervical cancer	Visual Inspection with Acetic Acid	3	1.15
	Pap Smear	3	1.15
	I do not know	254	97.7

### Practice of cervical cancer screening

Only 3.8% of respondents were screened for cervical cancer. The majority (53.2%) of respondents said that the barrier to having cervical cancer screening was a lack of health education programs to promote screening and 11.6% revealed that the screening place is too far from the place where they live (Table 4).

**Table 4:** Practice of cervical cancer screening among reproductive age group women in districts of Gurage zone, Southern Ethiopia, 2019.

Variables	Category	Frequency	Percentage
Have you ever screened for cancer of the cervix	Yes	10	3.8
	No	250	96.2
If yes how many times you screened	Once	10	100
When was the last time you screened	Within the past three years	6	60.0
	Before three years ago	4	40.0
What were the barriers to having cervical cancer screening	The screening places are too far from where I live	29	11.6
	I think the price is expensive	75	30.0
	There are no health education programs to promote screening	133	53.2
	I am afraid a screening test would reveal cervical cancer positive.	13	5.2
Do you have a plan to screen cervical cancer	Yes	91	35.0
	No	169	65.0

### Lifestyle and sexually related characteristics of the respondents

Of the total respondents, 29 (11.2%) of them had a history of smoking. About 12.3% of respondents had a history of sexually transmitted diseases in their parents and 43.1% of the respondents had a history of more than one sexual partner in their life (Table 5).

**Table 5:** Lifestyle and sexually related characteristics of respondents among reproductive age group women in districts of Gurage Zone, Southern Ethiopia, 2019.

Variables	Category	Frequency	Percentage
History of smoking	Yes	29	11.2
	No	231	88.8
Have you ever been told you that you had a pelvic infection or treated by health professionals?	Yes	60	23.1
	No	200	76.9
Do you have history of sexually transmitted infection in your lifetime?	Yes	28	10.8
	No	232	89.2
Does your partner ever have a history of sexually transmitted diseases?	Yes	32	12.3
	No	228	87.7
Number of sexual partners	1	148	56.9
	>1	112	43.1

### Factors associated with knowledge of cervical cancer screening

The result of the study showed that; illiterate/uneducated respondents were 15 (AOR = 15.5, 95%CI; 3.82–62.967) times higher odds of poor knowledge about cervical cancer screening than those respondents who had an educational status of college and above. Respondents who have plans to screen cervical cancer, family history of cervical cancer, menarche age, and age at first sex were significantly associated with good knowledge. Having plans to screen cervical cancer (AOR = 0.352, 95%CI;.175-.710), family history of cervical cancer (AOR = 14.158, 95%CI;3.88–51.7) menarche age between 9–12 years (AOR = 2.63, 95%CI; 1.28–5.37), and age at first sex between 15–17 years (AOR = 3.17, 95%CI; 1.283–7.837) were approximately 0.352 times, 14 times, 2.63 times and 3.17 times respectively more likely to have a good knowledge (Table 6).

**Table 6:** Factors affecting knowledge of cervical cancer screening among reproductive age group women in districts of Gurage zone, Southern Ethiopia, 2019.

Variables	Category	Knowledge		OR (95% CI)	
		Good	Poor	COR	AOR
Educational status	Illiterate/uneducated	3(6.3)	45(93.7)	7.808(2.28–26.79)	15.5(3.82–62.967)*
	Primary	8(23.5)	26(76.5)	1.692(.699–4.095)	2.084(.733–5.925)
	Secondary	19(28.4)	48(71.6)	1.315(.680–2.545)	1.648(.707–3.842)
	College & above	38(34.2)	73(65.8)	1	1
Utilization of family planning	Yes	39(31.7)	84(68.3)	1	1
	No	27(22.0)	96(78.0)	1.65(0.93–2.92)	1.078(.535–2.171)
History of abortion	Yes	15(33.3)	30(66.7)	1	1
	No	53(24.7)	162(75.3)	1.53(0.76–3)	.953(.379–2.396)
Family history of cervical cancer	Yes	15(68.2)	7(31.8)	7.5(2.9–19.3)	14.158(3.88–51.7)*
	No	53(22.3)	185(77.7)	1	1
Plan to screen cervical cancer	Yes	32(35.2)	59(64.8)	0.499(0.28–0.88)	.352(.175-.710)*
	No	36(21.3)	133(78.7)	1	1
Smoking status	Yes	13(44.8)	16(55.2)	0.3846(0.17–0.85)	.626(.215–1.823)
	No	55(23.8)	176(76.2)	1	1
Presence of sexually transmitted disease in partner	Yes	12(37.5)	20(62.5)	1	1
	No	56(24.6)	172(75.4)	1.84(0.848–4.007)	2.345(.826–6.658)
Age of Menarche	13–15	32(21.5)	117(78.5)	1	1
	9–12	36(32.4)	75(67.6)	1.755(1.005–3.07)	2.63(1.28–5.37)*
Age at first sex	15–17	15(42.9)	20(57.1)	2.459(1.16–5.214)	3.17(1.283–7.837)*
	18–22	43(23.4)	141(76.6)	1.781(0.62–5.158)	2.031(.593–6.956)
	23–26	8(29.6)	19(70.4)	1	1

*Statistically significant.

^**1**^Reference.

### Factors associated with the practice of cervical cancer screening

Age at first sex, having information about cervical cancer and having multiple sexual partners were significantly associated with the practice of cervical cancer screening.

Age at first sex between 15–17 years (AOR = 6.05, 95%CI 1.167–31.36), having information about cervical cancer (AOR = 10.2, 95% CI 1.9–96.4), more than one sexual partner (AOR = 3.96, 95%CI 1.48–10.58) were 6 times, 10.2 times and 3.96 times more likely to have practiced cervical cancer screening (Table 7).

**Table 7:** Factors affecting the practice of cervical cancer screening among reproductive age group women in districts of Gurage zone, Southern Ethiopia, 2019.

Variables	Category	Practice		OR(95% CI)	
		Yes	No	COR	AOR
Age	15–24	6(25.0)	18(75.0)	1	1
	>24	31(13.1)	205(86.9)	.386(.077–1.931)	2.49(0.68–9.12)
Age at first sex	15–17	5(14.3)	30(85.7)	10.06(2.28–44.29)	6.05(1.167–31.36)*
	18–22	22(12.0)	162(88.0)	2.083(.37–11.676)	1.139(.156–8.297)
	≥23	7(25.9)	20(74.1)	1	1
Post-coital bleeding	Yes	8(25.0)	24(75.0)	1	1
	No	29(12.7)	199(87.3)	2.3(0.94–5.66)	1.9(0.65–5.5)
History of abortion	Yes	11(24.4)	34(75.6)	1	1
	No	26(12.1)	189(87.9)	3.398(.918–12.58)	2(0.78–5.46)
Having information about cervical cancer	Yes	36(18.8)	156(81.2)	5.1(2–42)	10.2(1.9–96.4)*
	No	5(7.4)	63(92.6)	1	1
Knowledge of cervical cancer screening	Yes	20(29.4)	48(70.6)	1	1
	No	17(8.9)	175(91.1)	7.46(2.8–19.84)	2.76(0.9–8.34)
Pelvic infection	Yes	2(3.3)	58(96.7)	1	1
	No	8(4.0)	192(96.0)	1.75(0.82–3.74)	0.69(0.27–1.78)
Partner’s sexually transmitted disease	Yes	3(9.4)	29(90.6)	1	1
	No	7(3.1)	221(96.9)	1.85(0.74–4.65)	1.62(0.55–4.74)
Number of sexual partner	1	6(4.1)	142(95.9)	1	1
	>1	4(3.6)	108(96.4)	2.67(1.2–5.9)	3.96(1.48–10.58)*

*Statistically significant.

^**1**^Reference.

## Discussion

This study was aimed to assess the knowledge and practice of cervical cancer screening and associated factors among reproductive age group women in districts of Gurage zone. The study showed that 83.8% of women have heard about cervical cancer. This is consistent with the study done in Gonder town, North West Ethiopia (78.7%), and Hawassa University Medical and Health Sciences female students (76.8%) [5, 14]. This result is higher than the findings in Butajira town (47.6%), Wollega University female students (54.4%), Wolaita zone hospitals (43.1%), Addis Ababa government hospitals (60.8%), Tikur Anbesa Hospital, Ethiopia (21.7%), Nigeria (52.8%), and Cameroon (28%) [5, 13, 15–20]. However, this finding is lower than the study done among female medical students of Crimea State Medical University in Ukraine (80%) [21]. This difference might be due to that this study was done in areas where the level of awareness is lower and another possibility might be due to the difference in respondents. On the other hand, only 2.3% of the respondents knew cervical cancer screening methods. This result is lower than studies done in Hawassa University Medical and Health Sciences female students, Crimea State Medical University female medical students, and Bhutan University graduates female students [5, 21, 22]. This might be due to the difference like the population studied; as this study is conducted on the community therefore their level of knowledge might not be comparable with medical university students. Surprisingly, 99.6% of the respondents had a low level of knowledge about the HPV vaccine as a prevention method. Thus, attention should be given to all reproductive age groups women to increase their knowledge level about cervical cancer screening.

The study found that the level of knowledge about cervical cancer screening was 26.2% with 95% CI (20.8–31.2). This finding is consistent with a study done in Gondar (31%), Addis Ababa (27%), and Butajira town (21%) [13, 14, 17]. Whereas the finding is slightly higher than studies done in Tanzania (19.2%) and Cameroon (3.6%) [20, 23]. This difference might be explained due to the difference in the background of the respondents, and the difference in the study period as these days better attention has been given to cancer of the cervix.

The finding of this study revealed that 3.8% of respondents had practiced about cervical cancer screening with 95% CI (1.5–6.5). This finding is lower than the study done in, Butajira town, Addis Ababa Ethiopia, Tanzania, and Kenya which showed that 15.1%, 21.9%, 14%, and 22% of respondents had practiced cervical cancer screening respectively [13, 17, 23, 24]. The difference might be due to a lack of adequate information about the severity of cervical cancer national policy in the rural community of Ethiopia. On the other hand, it might be due to limited access or lack of screening services, and a low level of knowledge among respondents.

Uneducated/illiterate respondents were 15 times less likely to have good knowledge of cervical cancer screening than those who were educated. This finding is also supported by the study done in Portland Jamaica, and Mexico [25, 26]. This might be due to that uneducated respondents may not have better information about cervical cancer screening.

Respondents who had a history of cervical cancer in the family were 14 times more likely to have good knowledge about cervical cancer screening than their counterparts. This finding was similar to the study done in hosanna Ethiopia [27]. This might be because those families who had exposure to cervical cancer can easily disseminate any clinical symptoms and adverse effects related information to their family.

Those respondents who hadn’t planned to screen for cervical cancer were 0.35 times less likely had good knowledge then respondents who had planned. This finding was consistent with the study done in Kenya [24]. Respondents who had information about cervical cancer were 10.2 times more likely to practice cervical cancer screening than their counterparts. This might be due to that those respondents who had awareness of cervical screening might enforce them to visit health institutions.

Respondents who had a history of multiple sexual intercourses were 3.96 times more likely practicing of cervical cancer screening. This result is similar to the study done in Hawassa Ethiopia [5]. This might be due to those respondents who had multiple sexual partners might seek medical care for other reasons like STD-related symptoms, along with this they might get universal screening upon institutional visit.

### Limitation

This study involves a small sample which might lead to statistical imprecision. The other limitation might be the nature of study design that may not show the cause and effect relationship.

## Conclusion

This study found that the knowledge and practice of cervical cancer screening are low. Educational status, family history of cervical cancer, having plans to screen for cervical cancer, age at first sex and age of menarche were factors that affect knowledge of cervical cancer screening whereas early age at first sex, having information about cervical cancer, and multiple sexual partners were factors that affect the practice of cervical cancer screening. The finding indicates that an urgent intervention should be needed from the local government and all concerned bodies to increase awareness and practice of cervical cancer screening services.

## Supporting information

S1 Fig Knowledge of respondents about cervical cancer screening. (DOCX)

S1 File English version questionnaire. (PDF)

S2 File Amharic version questionnaire. (PDF)

S1 Dataset SPSS data set. (SAV)

## Abbreviations

AOR: Adjusted Odd Ratio
CI: Confidence Interval
COR: Cruds Odds Ratio
HPV: Human Papilloma Virus
SPSS: Statistical Package for Social Sciences
VIA: Visual Inspection with Acetic Acid
