# Supplementary material for: Knowledge and practice of cervical cancer screening and associated factors among reproductive age group women in districts of Gurage zone, Southern Ethiopia. A cross-sectional study
Source: PLoS One. 2020 Sep 18;15(9):e0238869. doi: 10.1371/journal.pone.0238869 (PMC7500695; doi:10.1371/journal.pone.0238869)
Supplement: S1 Fig — (DOCX) [file pone.0238869.s001.docx]

**S1 Fig: Knowledge of respondents about cervical cancer screening.**
