# Supplementary material for: Knowledge and practice of cervical cancer screening and associated factors among reproductive age group women in districts of Gurage zone, Southern Ethiopia. A cross-sectional study
Source: PLoS One. 2020 Sep 18;15(9):e0238869. doi: 10.1371/journal.pone.0238869 (PMC7500695; doi:10.1371/journal.pone.0238869)
Supplement: S1 File — (PDF) [file pone.0238869.s002.pdf]

Appendix

English version participant information sheet

Greeting: Good morning/afternoon!!

My name is………………………………I am working as a data collector for the study entitled Knowledge and practice of cervical cancer screening and associated factors among reproductive age group women in districts of Gurage zone, Southern Ethiopia, 2019. Now you are randomly selected for this study and I kindly request you to lend me your attention to explain about the study and being selected as the study participants.

The Study Title: Knowledge and practice of cervical cancer screening and associated factors among reproductive age group women in districts of Gurage zone, Southern Ethiopia, 2019

Name of the organization: Wolkite University, College of Medicine and Health Sciences, Department of Midwifery.

Introduction: Information sheet and consent form is prepared for reproductive age group who will be volunteer to participate in research project, community based cross-sectional study will be conducted to assess cervical screening status at this community.

Purpose/aim of the study:

The finding of this study can be of a paramount importance for the Zonal Health Bureau and other concerned bodies to plan different programs for the purpose of decreasing maternal morbidity and mortality related to cervical cancer by knowing the gaps.

Procedure and duration:

To assess women's cervical cancer screening status, you are invited to take part in this project. If you are willing to participate in this project, you need to understand and say "yes" on the agreement form. Then after, I will interview you and fill using structured questionnaires. There are 37 Questionnaires it will take about 30 minute to answer it, so I kindly request you to spare me this time for the interview.

Risk/ Discomfort: The risk of being participating in this study is very minimal, but you may feel some discomfort especially on spending time about 30 Minutes. We hope you will participate in

the study for the sake of the Benefit of the research result. I am sure there is no risk in participating in this research project.

Benefits/incentives, payments: There will not be any direct payment for participating in this study. But the findings will help us to identify the gap and take the appropriate intervention by the authorized stakeholders. You will not be provided any incentive or payment to take part in this project.

Confidentiality: The information you will provide us will be confidential. There will be no information that will identify you in particular. The findings of the study will be general for the study population and will not reflect any thing particular of individual persons. The questionnaire will be coded to exclude showing names. No reference will be made in oral or written reports that could link participants to the research.

Right to refuse or withdraw: Participation for this study is fully voluntary. You have the right to declare participate or not to participate in this study. If you decide to participate, you have the right to withdraw from the study at any time and this will not label you for any loss of benefits which you otherwise are entitled you do not have answer any questions that you do not want to answer.

Contact Address:

If there are any questions or enquiries any time about the study or the procedure, please **contact**:

Mobile Phone**:** 0921788975 or **Email Address:**danieladane178@yahoo.com

Appendix 1: English version participant consent form

I have read/ was read to me the participant information sheet. I have clearly understood the propose of the research, the procedures, the risk and benefits, issues of confidentiality, the rights of participating and the contact address of any queries. I have been given the opportunity to ask questions for the things that may have been unclear. I was informed that I have the right to withdraw from the study at any time or not to answer any questions that I do not want. Therefore, I declare my voluntary consent to participate in this study.

Volunteer to participate: **Yes** ☐ **No** ☐

If participant does not agree to be interviewed thanks him/her and go to the next participant.

If respondent say **yes** continue.

Appendix 3: English version questionnaire

This is a data collection format to assess knowledge and practice of cervical cancer screening and associated factors among reproductive age group women in Gurage zone, Southern Ethiopia, 2019.

Name of Data collector: ---------------------------------- Qualification: ------------------------

Time Interview Started: Hour: _____ Minute: _____ Ended: Hour: _____ Minute: _____

Questionnaire No____________________________

Date ______/_____/____ ____E.C. signature___________

Data Collector agreement

"I certify that I have filled the questionnaire in accordance with the training that is given to me and instructions stated in it. I have confirmed that the information in it is correct."

Signature _______________Date______/_____/____ ____E.C

Name of kebeles _________________Code ___________________

Checked by supervisor for completeness: Supervisor Name ______________signature____

Date___/___/__E.C.

Table 1: questionnaires to assess Knowledge and practice of cervical cancer screening and associated factors among reproductive age group women in districts of Gurage zone, Southern Ethiopia, 2019.

**Circle and write the appropriate response accordingly**			
**PART I:- Socio-demographic characteristics of respondents**			
S. No	Questions	Response	Remark
001	Age group of participants	Age in completed years________	
002	Marital status	1. Single<br>2. Married<br>3. Divorced<br>4. Widowed<br>5. Other (specify)	
003	Ethnicity	1. Gurage<br>2. Oromo<br>3. Amhara<br>4. Kembata<br>5. Others (specify)	
004	Religion	1. Catholic<br>2. Protestant<br>3. Muslim<br>4. Orthodox	

005	Work	1. Farmer<br>2. House wife<br>3. Government Employee<br>4. Merchant<br>5. Others(specify)	
006	Monthly income	Ethiopian birr---------------------	
007	Educational status	1. Educated<br>2. Uneducated	If Uneducated skip to Question No 009
008	Educational level	1. Primary education<br>2. Secondary education<br>3. Diploma<br>4. Degree and above	
**Part II:- Reproductive characteristics of respondents**			
009	Age of Menarche	Age in completed years-----------	
010	Sex condition	1. Yes<br>2. No	If answer is No skip to Q No 12
011	Age at first sex	In completed years-------	
012	Contraceptive history	1. Yes<br>2. No	If answer is No skip to Q No 14
013	What type of family planning used? (more than one answer is possible)	1. Pills<br>2. Injectables<br>3. Implants<br>4. Intrauterine contraceptive device<br>5. Condom	
014	Nature of menses	1. Regular<br>2. Sometimes irregular<br>3. Always irregular<br>4. No menses	
015	History of abortion	1. Yes<br>2. No	
016	Family history of cervical cancer	1. Yes<br>2. No	
**Part III:- Knowledge related questions**			
017	Have you heard of cervical cancer?	1. Yes<br>2. No	If answer is No skip to Q No 19
018	Source of information? (more than one answer is possible)	1. From media<br>2. Broachers and printed materials<br>3. Health workers<br>4. Family, friends, neighbors<br>5. Others (please specify)------	
019	What cervical cancer symptoms do you know? (more than one answer is possible)	1. Vaginal bleeding<br>2. Vaginal foul-smelling discharge<br>3. I don't know<br>4. Post-coital bleeding)	
020	What are the risk factors for cancer of the	1. Having multiple sexual partners	

	cervix? (more than one answer is possible)	2. Early sexual intercourse<br>3. Cigarette smoking<br>4. Acquiring Human Papilloma Virus<br>5. I do not know<br>6. Others(specify)--------	
021	How can cervical cancer be prevented? (more than one answer is possible)	1. Through minimizing multiple sexual partners<br>2. Through avoiding early sexual intercourse<br>3. Through abandon smoking<br>4. Through vaccination of Human Papilloma Virus<br>5. I do not know	
022	Can cancer of the cervix be cured at the first stage	1. Yes<br>2. No<br>3. I do not know	
023	How can someone with cancer of the cervix be treated? (more than one answer is possible)	1. Herbal remedies<br>2. Surgery<br>3. Specific drugs are given by the hospital<br>4. Radiotherapy<br>5. I do not know	
024	How expensive do you think cancer of the cervix treatment is in this country is in this country	1. It is free of charge<br>2. It is reasonably priced<br>3. Moderately expensive<br>4. It is very expensive<br>5. I do not know	
025	Do you know that there is cervical cancer screening?	1. Yes<br>2. No	
026	How frequent is screening for cervical cancer performed?	1. Once every year<br>2. Once every three years<br>3. Once every 5 years<br>4. 1 do not know	
027	Can you mention any of the procedures used in screening for cervical cancer?	1. Pap Smear<br>2. I do not know<br>3. Visual Inspection with Acetic Acid<br>4. Others (specify)-------	
**Part IV:- Practice related questions**			
028	Have you ever screened for cancer of the cervix?	1. Yes<br>2. No	If no skip to Q No 031
029	If yes how many times you screened?	1. Once<br>2. More than once	
030	When was the last time you screened?	1. Within the past three years<br>2. Before three years ago	

031	What were barriers to have cervical cancer screening?	1. The screening places are too far from where I live<br>2. I think the price is expensive<br>3. There are no health education programs to promote screening<br>4. I am afraid a screening test would reveal cervical cancer positive.<br>5. Others (**Specify**)…………..	
032	Do you have a future plan to screen Cervical cancer ?	1. Yes<br>2. No	

Part V:- Lifestyle and sexual related to behavior questions

033	Have you ever smoked?	1. Yes<br>2. No	
034	Have you ever been told you that you had a pelvic infection or treated by health professionals?	1. Yes<br>2. No	
035	Have you had a sexual transmitted infection in your life time?	1. Yes<br>2. No	
036	Does your partner ever have history of STD?	1. Yes<br>2. No	
037	How many sexual partners have you had in your life time?	--------------------	
