# Supplementary material for: Knowledge and practice of cervical cancer screening and associated factors among reproductive age group women in districts of Gurage zone, Southern Ethiopia. A cross-sectional study
Source: PLoS One. 2020 Sep 18;15(9):e0238869. doi: 10.1371/journal.pone.0238869 (PMC7500695; doi:10.1371/journal.pone.0238869)
Supplement: S2 File — (PDF) [file pone.0238869.s003.pdf]

1 ቅጥያ

I. **መጠይቆች በአማርኛ**

ቅጥያ 1 የተሳታፊዎች መረጃ

እንደምን አደሩ/ዋሉ !!!

ስሜ——————————————እባላለሁኝ።

በወልቂጤ ዩኒቨርሲቲ በህክምናና ጤና ሳይንስ ኮሌጅ በሚድዋይፈሪት/ት ክፍል በሚጣና ጥናት ላይ መረጃ ሰብሳቢ ሆኜ እየሰራሁ ነው።እርስዎም በዚህ ጥናት ላይ እንዲሳተፉ ስለተጋበዙ የእርስዎን ታማኝ እና ቀና የሆነ ትብብር ለጥያቄዎቹ መልስ እንፈልጋለን።

የጥናቱርዕሰ:በደ/ብ/ብ/ሕክልላዊ መንግስት በጉራጌዞን በሚገኙ ወረዳዎች ባሉ ሴቶች ዘንድ ያለውን የማህጸን ካንሰር እሰክሪኒንግ አገልግሎት እዉቀት፣ተግባር እና ተያያዥ ምክኒያቶችን ላማወቅ ነው።

የተቋሙሰም :ወልቄጤ ዮኒቨርስቲ, የጤና ሳይንሰ ኮሌጅ, የሚድዋይፍሪ ት/ት ክፍል

የሰፖንሰር ሰም :ወልቄጤ ዮኒቨርስቲ

መግቢያ: በዚህ ጥናት ላይ ፈቃደኛ ለሆኑ መውለድ ለሚችሉ ሴቶች የመረጃ እና የፈቃደኝነትቅጽ ተዘጋጀቷል።በዚህማህበረሰብውስጥያለውንየማህጸንካንሰርእሰክሪኒንግነባራዊሁኔታለመገምገምእንዲረዳ ማህበረሰብተኮርጥናትይካሄዳል።

የጥናቱአላማ:የዚህጥናትውጤትየዞኑጤናቢሮእናሌሎችየሚመለከታቸውአካላትከማህፀእካንሰርጋርየተያ ያዙየእናቶችጉዳትእናሞትንለሚያቅዷቸውመርሀግብሮችመሰካትከፍተኛጠቀሜታአለው።

ቅደም ተከተል እና የሚፈጀው ጊዜ የሴቶች የማህፀን ካንሰር ስክሪኒግ ሁኔታ ለመገምገም በሚደረገው ጥናት ላይ እንዲሳተፉ ከተጋበዙ በኋላ ፈቃደኛ ከሆኑ የስምምነት ቅፅላይ አዎ የሚለውን ምርጫ የወስዳሉ። በመቀጠል መጠይቁን እያካሄድን የተዘጋጁትን መጠይቆች ምርጫ ይወስዳሉ። የመጠይቆቹ ብዛት **37** ሲሆኑ ቃለመጠየቁን ለማጠናቀቅ 30 ደቂቃ ያስፈልጋል ስለዚህ ይህን ሰዓት እንድጠቀም እንዲፈቅዱልኝ ስንል በትህትና እጠይቃለሁ።

የሚፈጠርችግር/አለመመቸት፡ የጊዜው 30 ደቂቃ ለዚህ ጥናት መጠቀምዎ ካለተመቸዎ በቀር እዚህ ጥናት ላይ መሳተፉዎ ምንም አይነት ችግር እንደማያመጣ በእርግጠኝነት አስረግጬ መናገር እፈልጋለሁ። ለጥናቱ የተሳካ ዉጤት ሲሉ ለመሳተፍ ፈቃደኛ እንደሚሆኑ ተስፋ እናደርጋለን።

ማበረታቻ/ክፍያ፡ዕዚህጥናትላይበመሳተፈዎበቀጥታየሚያገኙትማበረታቻወይምክፈያባይኖርምየጥናቱ ውጤትግንያለውንክፍተትለይቶበማውጣትባለድርሻአካላትለችግሩመፍትሔውንእንዲፈጥሩለማድለግያስ ችላል። ስለዚህ ጥናቱ ላይ ስለተሳተፉየሚያገኙትክፍያወይምማበረታቻአይኖርም።

ሚስጥራዊነት፡ የሚሰጠን መረጃ ምስጥራዊነት የተጠበቀ ከመሆኑም በላይ እርሶዎን ነጥሎ የሚጠቀም ምንም አይነት መረጃ አይኖርም የጥናቱ ውጤቶች ከጠቅላላው ህዝብጋር እንጂ ከግለሰብጋር ለብቻው የተያያዘነገር አይኖራቸውም መጠይቆቹ ስም እንዳያስፈልጋቸው ተደርገው የተዘጋጁ ከመሆናቸው በላይ ተሳታፊ ግለሰብን ከጥናቱጋር ሊያገናኙ የሚችሉ የቃልም ይሁን የተፃፈ ማጣቀሻዎች አይኖሩም።

የመቃወም ወይም የማቋረጥ መብት፡-ጥናቱ ላይ ለመሳተፍ የተሳታፊው ሙሉ ፍቃድ አስፈላጊ እንደመሆኑ ግለሰቡ የመሳተፍም ሆነ ያለመሳተፍ ሙሉ መብት አላቸው ከተሳተፉም ብኋላም ቢሆን በፈለጉት ሰአት የማቋረጥ እና መመለስ የማይፈልጉትን ጥያቄ ያለመመለስ ሙሉመብት አላቸው።

አድራሻ፤ ስለጥናቱ ምንም ዓይነት ጥያቄ ካለ በዚህ ያሳውቁን

Mobile phone : +251921788975 or Email: danieladane178@yahoo.com

ቅጥያ 2 : የተሳታፍየፍቃድቅጽ የተሳታፊ መረጃ ቅጹን አንብቤያለሁ ወይም ተነቦልኛል: ስለጥናቱ ዓላማ፣ ቅድም ተከተል፣ ጉዳትናጥቅሞች፣ ምስጥራዊነት፣ የተሳትፎ መብትእናየግንኙነት አድራሻዎች በግልጽ ተረድቻለው፣ግልጽ ያልሆኑልኝን ነግሮች እንድጠይቅም እድሉተሰቶኛል : : በፈለኩት ሠዓት ጥናቱን ማቋረጥ እና መመለስ ማልፈልጋቸውን ጥያቄዎችም አለመመለስ ዕንደምችል አሳውቀውኛል : : ስለሆነም በዚህጥናትላይ ለመሳተፍያለኝን ፍቃደኝነት አሳውቄአለሁ::ግለሰቡ ለቃለመጠይቁ ፈቃደኛ ካልሆኑ አመስግነን ወደ ቃጣይ ተሳታፊ ይታለፋል።

ፈቀዳኛኖት አዎ ☐ አይደለሁም ☐

ግለሰቡ እሽካለ ጥናቱ ይቀጥላል።

መጠይቆች በአማርኛ ይህ ይዘታ——————————ለመገምገም ተዘጋጀ የመረጃ መሰብሰቢያ ነው።

የመረጃው ሠብሳቢው/ዎ ስም ________________________ልዩ ብቃት____________________

ቃለመጠየቁ የጀመረበት ጊዜ፣ሠዓት __________________ደቂቃ___________________

ቃለመጠየቁየተጠናቀቀበትጊዜ፣ሠዓት_________________ደቂቃ__________________

መጠይቅ ቁጥር________________

የመረጃ ሠብሳቢ ስምምነት : መጠይቆቹን የሞላሁት በተሰጠኝ ስልጠና እና መመሪያዎች መሠረት መሆኑ እና ያሉት መረጃዎች ትክክለኛ መሆናቸውን አረጋግጣለሁ።

ፊርማ____________ቀን_____/______/_______E.C

ነተቆጣጣሪው ተረጋግጧል። የተቆጣጣሪው ስም________ፊርማ__________ቀን______/_____/____ ዓ.ም

ሰንተረጅ 1፡ በጉራጌዞን በሚገኙ ወረዳዎች ባሉ ሴቶች ዘንድ ያለውን የማህጸን ካንሰር እስክሪኒንግ አገልግሎት እዉቀት፣ተግባር እና ተያያዥ ምክኒያቶችን 2011 ዓ.ም

**ክፍል 1፡ ማህበራዊና የስነህዝብ ጉዳዮች**		
001	እድሜሽ ስንት ነው?	---------------------
002	የትዳርሽ ሁኔታ እንዴት ነው?	1. ያላገባች<br>2. ያገባች<br>3. የፋታች<br>4. ባሏ የሞተባት
003	ጠቅላላ የወር ገቢዎ ስንት ነው?	---------------------
004	የዘርሀረግ/ጎሳ ?	1.ጉራጌ<br>2. ኦሮሞ<br>3. አማራ<br>4. ከምባታ<br>5. ሌላ ካለ ይገለፅ---
005	የትምህርት ሁኔታ ?	1. የተማረ<br>2. ያልተማረ
006	የትምህርት ደረጃዎ ?	1. የ መጀመሪያ ደረጃ<br>2. ሁለተኛ ደረጃ<br>3. ዲፕሎማ<br>4. ዲግሪ እና ከሀያ በላይ
007	የስራሁ ኔታዎ ?	1. ግብርና<br>2. የ ቤት እመቤት<br>3. ተቀጣሪ<br>4. ነጋዴ<br>5. ሌላ ካለ ይገለፅ--
008	ሃይማኖትሽ ምንድን ነው?	1.ኦርቶዶክስ<br>2.ፕሮቴስታንት<br>3. ሙስሊም<br>4. ካቶሊክ<br>5 ሌላ ካለ ይገለፅ---
**ክፍል 2:- ስላ ስነተዋልዶ ባህሪያት**		
009	በመጀመሪያ ጊዜ የወር አበባ የዪት በስንት አድሜዎ ነበር	
010	የግብረ ስጋግንኙነት ፈፀመው ያውቃሉ?	1. አዎ 2.የለም

011	መልስዎ አዎ ከሆነ ለመጀመሪያ ጊዜ የ ፈፀሙት በስንት አመትዎ ነው?	————
012	የወሊድ መከላከያ ተጠቅመው ያውቃሉ ?	1 . አ ዎ<br>2 . የለም
013	የወሊድ መከላከያ እየተጠቀሙ ከሆነ ወይም ከነበር የትኛውን አይነት ነው የሚጠቀሙት?	1. የሚዋጥ ፒል<br>2. በመርፌ የሚሰጥ<br>3. በክንድ የሚቀበረውን<br>4.በማህጸን ውስጥ የሚቀበር<br>5. ኮንዶም
014	የወር አበባዎ ዑደት እንዴት ነው?	1. በየወሩ በትክክል ይመጣል<br>2. አንዳንዴ ትዛባል<br>3. ብዙ ግዜ ይዛባል<br>4. የወር አበባ አላይም
015	ውርጃ ኖሮት ያውቃሉ?	1. አዎ<br>2. አያውቅም
016	በቤተሰብ የማህጸን ካንሰር ያለበት ሰው አለ?	1. አዎ<br>2. የለም
**ክፍል 3፡ የማህፀንካንሰርላይያላቸውእውቀት(ከአንድ መልስ በላይ መመለስ ይቻለል)**		
017	የማህፀን ካንሰር ሲባል ሰምተው ያውቃሉ?	1. አዎ<br>2. የለም
018	ሰለ ማህፀን ካንሰር ለመጀመሪያ ጊዜ የሰሙት የት ነው ?	1. መገናኛ ብዙሃን<br>2. ማስታወቂያ እና ሌሎች የ ህትመት መሳሪያዎች<br>3. ጤና ሠራተኞች<br>4. በተሰብ : ጓደኞች : ጎረቤት እና ባልደረባዎች<br>5. ሌላ ካለ ይገለፅ---
019	የማህፀን ካንሰር ምልክቶች ምንድን ናቸው?	1. የ ብልት መደማት<br>2. ከብልት የሚወጡ ጠረን ያላቸው ፈሳሾች<br>3. አላውቅም<br>4. ከገንኙነት በሆላ ህመም
020	የማህፀን ካንሰርን የሚያመጡ መንስኤዎች ምንድን ናቸው?	1. ብዙ የወሲብ ጓደኞች መያዝ<br>2. ያለ ዕድሜ ወሲብ መፈፀም<br>3. ሲጋራ ማጨስ<br>4. HPV ቫይረስ<br>5. ሌላ ካለ ይገለፅ---
021	የማህፀን ካንሰርን እንዴት መከላከል እንችላለን?	1. የ ወሲብ ጓደኛ አለማብዛት<br>2. ያለ ዕድሜ ወሲብ አለመፈፀም<br>3. ሲጋራ አለማጨስ<br>4. HPV ክትባት በመከተብ<br>5. አላውቅም<br>6. ሌላ ካለ ይገለፅ---

022	የማህፀን ካንሰርን በመጀመሪያ ደረጃዎች መዳን ይችላል?	1. አዎ<br>2. የለም<br>3. አላውቅም
023	የማህጸን ካንሰር የያዘው ሰው እንዴት ነው ሊድን የሚችለው?	1.የክትትል ህክምና<br>2. ቀዶ ጥገና<br>3. ሆስፒታል በሚያዘው መድሀኒቶች<br>4.ራጅ<br>5.አላውቅም 6. ሌላ
024	እዚህ ሀገር ላይ የማህጸን ካንሰር ህክምና ምን ያህል ውድ ነው ብለው ያስባሏ?	1.ነጻ ነው<br>2.ዋጋው የተመጣጠነ ነው<br>3. በመጠኑ ውድ ነው<br>4. በጣም ውድ ነው<br>5. አላውቅም
025	የማህጸን ካንሰር ምርመራ እንዳለ ያውቃሉ?	1.አዎ 2. የለም
026	የማህጸን ካንሰር ምርመራ ምን ያክል ተደጋግሞ ይሰራል?	1.በአመት 1 ግዜ<br>2.በ3 ዓመት 1 ግዜ<br>3. በ5 ዓመት 1 ግዜ<br>4.ሌላ ካለ ይግለጹ
027	ለማህጸን ካንሰር ምርመራ የምንጠቀማቸው ቅድመ ተከተሎች የሚያውቋት አለ?	1.ቪዐይፒ<br>2. ፓፕ ስሚር 3 .ባዮፒሲ<br>4. አላቅም 5. ሌላ ካለ ይግለጹ
**ክፍል 4፡ የማህጸን ካንሰር ምርመራ የተመለከቱ ጥያቄዎች**		
028	ለማህጸን ካንሰር ምርመራ ተደርገው ያውቃሉ?	1. አው 2.አላቅም
029	መልስዎ አዎ ከሆነ ምን ያክል ጊዜ?	1. አንድ ግዜ<br>2. ከአንድ ግዜ በላይ
030	ለመጨረሻ ጊዜ ምርመራ የተደረጉት መቼ ነው?	1. ባለፉት 3 ዓመት ውስጥ 2.ከ 3 ዓመት በፍት
031	የማህጸን ካንሰር ምርመራ ማድረግ ያልቻሉበት ምክኒያት ምን ምን ናቸው?	1. ምርመራ የሚሰጥባቸው በታዎች ከመኖሪያ አካባቢ የራቁ ስለሆነ<br>2. የምርመራ ዋጋ ውድ ስለሚመስል<br>3.የማጸን ካንሰር ምርመራ ማድረግ የሚያስችሉና የሚያበረታቱ የጤና ፕሮግራሞች አለመኖር<br>4.ካንሰር አለብሽ የሚል የምርመራ ውጤት መስማት ስለምፈራ<br>5.ሌላ----
032	የማህጸን ጫፍ ካንሰርቅድመ ምርመራለማድረግ እቅድ አለዎት?	1.አዎ 2. የለም
**ክፍል 5፡ አኖኖር ሁኔታጋር ተያያዡ ምክኒያቶች**		
033	ሲጃራ አጬሰው ያውቃሉ?	1. አዎ 2. አላውቅም
034	የማህጸን ኢንፌክሽን አለበት ተብለው ወይም ታክመው ያውቃሉ?	1. አዎ 2. አላውቅም
035	የአባላዘር አለበዎት ተብለው ታክመው ያውቃሉ?	1. አዎ 2. አላውቅም
036	ባለቤትዎ ወይም የፍቅር ጓደኛዎ የአባላዘር በሽታ አለበት ተብለው ወይም ታክመው ያውቃሉ	1. አዎ 2. አያውቅም
037	እስካሁን ከስንት ወንዶች ጋር ግብረ ስጋ ግንኙነት አድርገው ያውቃሉ?	- - - - - - - - - - - - - - - -
